# Incidence and Risk Factors of Iatrogenic Retinal Breaks: 20-Gauge versus 25-Gauge Vitrectomy for Idiopathic Macular Hole Repair

**DOI:** 10.1155/2020/5085180

**Published:** 2020-02-13

**Authors:** Norio Fujiwara, Goji Tomita, Fumihiko Yagi

**Affiliations:** ^1^Hamadayama Fujiwara Eye Clinic, Tokyo, Japan; ^2^Department of Ophthalmology, Toho University Ohashi Medical Center, Tokyo, Japan

## Abstract

**Purpose:**

We compared the incidences of iatrogenic retinal breaks and postoperative retinal detachment between eyes that underwent 20-gauge vitrectomy and those that underwent 25-gauge vitrectomy for idiopathic macular hole repair.

**Methods:**

This retrospective nonrandomized consecutive observational case series included 185 eyes of 183 patients (130 eyes of 129 patients and 55 eyes of 54 patients in the 20- and 25-gauge groups, respectively). We assessed the relationship between the incidence of retinal breaks and postoperative retinal detachment and related this to posterior vitreous detachment and lattice degeneration.

**Results:**

The incidences of iatrogenic retinal breaks were 36.9% and 12.7% in the 20-gauge and 25-gauge groups, respectively. These groups did not differ in their respective frequencies of posterior vitreous detachment (the 20-gauge group: 31.5% and the 25-gauge group: 27.3%) and lattice degeneration (the 20-gauge group: 14.6% and the 25-gauge group: 7.3%). Among eyes without lattice degeneration, the 20-gauge group showed a higher incidence of iatrogenic retinal breaks than the 25-gauge group. However, among the eyes with lattice degeneration, the frequency of retinal breaks did not differ between the two surgery types, and four cases of postoperative retinal detachment were reported in both groups.

**Conclusions:**

The incidence of retinal breaks related to idiopathic macular hole surgery is higher among patients undergoing 20-gauge vitrectomy than among those undergoing 25-gauge vitrectomy. Posterior vitreous detachment and lattice degeneration are associated with considerably increased incidences of retinal break.

## 1. Introduction

The transconjunctival sutureless 25-gauge (G) pars plana vitrectomy system was first described by Fujii et al. [1] in 2002, and then refined by Eckardt [2] introduction of the 23 G system in 2005. In 2010, Oshima et al. [3] produced the 27 G system. These minimally invasive techniques result in superior wound-sealing and are now widely accepted by vitreoretinal surgeons.

Idiopathic macular holes (MHs) are common in the elderly, particularly women, and lead to loss of central vision, distortion of vision, and scotoma [4–6]. Recently, vitrectomy with internal limiting membrane (ILM) staining using indocyanine green [7], triamcinolone acetonide [8], and brilliant blue G [9] in combination with the inverted ILM flap technique [10] has proven to be an effective intervention for MHs.

Iatrogenic retinal breaks (RBs) are recognized as a serious potential complication of vitrectomy. If undetected or untreated, postoperative retinal detachment (RD) can occur, necessitating additional surgery and sometimes leading to permanent vision loss. The incidence of RBs in patients with MH is 0%–35.4% for 20 G surgery [11–16], 3.6%–18.3% for 23 G surgery [14, 17], and 17.3% for 25 G surgery [18]. Prior identification of eyes with an increased risk of intraoperative RB is important. The aims of this retrospective nonrandomized consecutive observational case study were twofold. First, we compared the incidences of RBs after conventional 20 G vitrectomy with those after sutureless 25 G vitrectomy, to assess surgical outcomes. Second, we used the same dataset to assess the influence of copathologies on the RB incidences following these two surgery types, using records for patients with and without posterior vitreous detachment (PVD) or lattice degeneration (LD).

## 2. Patients and Methods

This study was designed as a retrospective nonrandomized consecutive observational case series. To treat MH, 185 vitrectomy procedures were performed by a single surgeon (F. Y.) either with a 20 G instrument (130 eyes of 129 patients; stage 1: 1 eye, stage 2: 19 eyes, stage 3: 69 eyes, and stage 4: 41 eyes) or a 25 G instrument (55 eyes of 54 patients; stage 1: 1eye, stage 2: 10 eyes, stage 3: 29 eyes, and stage 4: 15 eyes) at the Department of Ophthalmology, Toho University Ohashi Medical Center, during a period from May 2006 to October 2018 (20 G, May 2006–July 2015; 25 G, July 2006–October 2018). For all patients, preoperative examinations included anterior segment examination and dilated fundus examination with a 90-diopter lens. In all eyes, the peripheral retina was examined, with scleral indentation at the completion of surgery to ascertain the presence or absence of RBs and LD.

Patients underwent a 3-port pars plana vitrectomy with either a 25 G or a 20 G instrument (Accuras® or Constellation®, Alcon Laboratories, Inc., Fort Worth, TX, USA). Core Vitrectomy was performed and, if not already present, PVD was induced. The aspiration pressure was set between 400 and 500 mmHg during creation of the PVD. The PVD was started from the optic disc and developed to the peripheral retina using the vitrectomy probe in cutter-off mode. Indocyanine green diluted to a concentration of 0.125% was used to aid ILM peeling. The PVD that extended as far as possible to the vitreous base was created, followed by vitreous shaving with scleral indentation. Any untreated RBs and LD were treated by endolaser photocoagulation followed by gas tamponade using 20% sulfur hexafluoride. The following seven items were retrospectively examined: (1) patient background; (2) incidence of iatrogenic RB; (3) frequency of postoperative RD; (4) frequency of preoperative PVD; (5) frequency of LD; (6) incidence of iatrogenic RBs with or without preoperative PVD and LD; and (7) initial and final MH closure rate.

The unpaired *t*-test and Fisher's exact probability test were used for analysis of numerical data. Statistical significance was set at *P* < 0.05. This study was approved by the Ethics Committee of Toho University Ohashi Medical Center (no. 20–22).

## 3. Results

### 3.1. Patient Background

This study included 56 eyes of 56 men and 74 eyes of 73 women in the 20 G group and 26 eyes of 25 men and 29 eyes of 29 women in the 25 G group. Neither the male-to-female ratio nor age at surgery (65.2 ± 8.6 y: mean ± standard deviation) differed between groups. In both the 20 G and 25 G groups, the majority of patients had phakic eyes before surgery. In the 20 G group, 116 of 120 phakic eyes underwent vitrectomy with phacoemulsification and intraocular lens implantation (PEA + IOL), and four underwent the same procedure, but with preservation of the natural clear lens. In the 25 G group, all 46 phakic eyes underwent vitrectomy with PEA + IOL. The proportion of vitrectomy with preservation of the natural clear lens was indistinguishable between the two surgery groups (Table 1).

Comparison of Incidence of Iatrogenic RBs, Postoperative RD, Preoperative PVD, and LDs between 20 G and 25 G groups.

The incidence of iatrogenic RBs during surgery was significantly higher in the 20 G than in the 25 G group. There was no oral dialysis. The incidences of postoperative RD, preoperative PVD, and LD did not differ between the two surgery groups. Table 1 gives values and statistics for all comparisons.

### 3.2. Comparison of Incidence of Iatrogenic RBs with or without Preoperative PVD and LD between 20 G and 25 G Groups

In the 20 G group, eyes without PVD were associated with a higher incidence of iatrogenic RBs than those with PVD (47.2% versus 14.6%, *P*=0.0007, Table 2). In the 25 G group, the incidence of iatrogenic RBs did not differ between eyes with and without PVD. Although the incidence of iatrogenic RBs in the 20 G group was significantly higher in the absence of PVD than that in the 25 G group (47.2% versus 10%, *P*=0.0001), it did not differ in the presence of PVD between the two surgery groups (in the 20 G group: 14.6% versus in the 25 G group: 20%) (Table 2).

In the 20 G group, the incidence of iatrogenic RBs was higher in the presence than in the absence of LD (78.9% and 29.7%, respectively, *P*=0.0001, Table 2). The same was true within the 25 G group (incidences of 75.0% and 7.8% RB in eyes with and without LD, respectively, *P*=0.002, Table 2). For eyes without LD, the 20 G group showed a significantly higher incidence of iatrogenic RBs than the 25 G group (*P*=0.021); however, the incidence of iatrogenic RBs did not differ between the two surgery groups for eyes with LD (Table 2).

### 3.3. MH Closure Rate

The initial MH closure rate was 93.8% (122 of 130 eyes) in the 20 G group and 94.5% (52 of 55 eyes) in the 25 G group. The difference between groups was not statistically significant. The final MH closure rate was 100% in both groups.

## 4. Discussion

Iatrogenic RBs are recognized as an important complication of MH surgery, leading to RD that severely compromises postoperative visual recovery. In 1991, Kelly and Wendel [11] reported that neither iatrogenic RBs nor postoperative RD developed in 52 eyes treated for MH; since then, MHs have benefited from the effective treatment developed by these authors. In 1993, Wendel et al. [12] reported that equal numbers of postoperative RBs and RDs (2, 1.2%) occurred separately among 170 treated eyes. In 1995, Sjaarda et al. [19] first determined the position of iatrogenic RBs associated with MH surgery, reporting that among 181 eyes treated for MH, iatrogenic RBs occurred in 10 (5.5%) and postoperative RDs in 2 (1.1%) eyes. Chung et al. [13] reported an incidence of iatrogenic RBs of 14.6% (20 of 137 eyes), with a postoperative RD incidence of 2.2% (3/174). Notably, these authors reported higher incidences of iatrogenic RBs in the presence of preoperative PVD than among eyes without PVD (12.7%, 19/105 eyes versus 3.1%, 1/32, respectively, *P*=0.008) [13].

Nakano et al. [15] compared 23 G and 20 G vitrectomies for MH and reported incidences of iatrogenic RBs of 3.6% (2/55) in the 23 G group and 20.8% (10/48) in the 20 G group (*P*=0.013). Although the RB incidences that we report are generally higher than this study (12.7% in the 25 G group and 36.9% in the 20 G group), our comparisons revealed the same trend: incidences of iatrogenic RBs are lower for vitrectomies performed with finer gauge instruments. Among eyes treated for MH, the incidence of iatrogenic RBs was 8.3% (2/24) and 32.3% (10/31) in the 23 G and 20 G groups, respectively [20]. These authors' results were similar to those of our study.

Only one of these previous reports [11–18] mentions the incidence of PVD, and none assessed the relationship between LD and successful vitrectomy. Therefore, we investigated the incidence of iatrogenic RBs in relation to two coexisting pathologies, PVD and LD. These occurred in 7.3%–14.6% of the eyes in our study, evenly distributed across the two vitrectomy groups. Sakamoto et al. [21] reported that among 382 eyes that underwent MH surgery, 25.7% (90/382) had preexisting PVD, and iatrogenic intraoperative RBs were detected in 20.9% (80/382). LD was detected in 5.5% (21/382) and 81.0% (17/21) of these had intraoperative RBs. Sakamoto et al. [21] have identified LD as a risk factor for intraoperative RBs and shown that RBs occurred significantly more often in eyes with untreated LD that in those with LD treated by laser photocoagulation.

The reason that the incidence of iatrogenic RBs in the 20 G group was significantly higher in the absence of PVD (47.2%) is unclear. We try to remove the vitreous thoroughly to avoid postoperative RD, which may be a reason. Sakamoto et al. [21] identified LD to be a significant risk factor for RBs during MH surgery and that preoperative laser photocoagulation for LD decreased the risk; however, prophylactic photocoagulation is unlikely to reduce the risk of spontaneous RD [22]. In this study, untreated RBs and LD were treated by endolaser photocoagulation, during surgery to avoid postoperative RD.

Suboptimal management of RBs may cause postoperative RD. If difficulties arise during cutting of the peripheral vitreous at the RB site, a scleral buckling procedure including an encircling band can be performed, particularly in phakic eyes. The gas tamponade integral to MH surgery may result in residual vitreous contraction, thereby causing new RBs and RD. Therefore, we also investigated whether the incidence of vitrectomy-related iatrogenic RBs is influenced by the presence of PVD and LD in the 20 G and 25 G groups.

The current study has a few limitations, including its retrospective nature, and different numbers of patients in the two groups (130 eyes of 129 patients: 20 G, and 55 eyes of 54 patients: 25 G). All surgical procedures were performed by a single experienced surgeon, and all relevant information, including surgical complications, was recorded in detail.

## 5. Conclusions

Sutureless 25 G vitrectomy was associated with a significantly lower incidence of RBs than 25 G vitrectomy, but this decreased incidence was restricted to eyes without coexistent pathologies. Within each technique, LD, and, to a lesser extent, PVD were associated with significantly increased incidences of RB.

## Figures and Tables

**Table 1:** Clinical characteristic of patients with idiopathic macular hole for 20-gauge and 25-gauge groups.

Patient/eye characteristic	20 gauge (130 eyes)	25 gauge (55 eyes)	*P* value
Age at surgery (y)	65.3 ± 8.3^*∗*^	65.0 ± 9.3^*∗*^	NS^†^
Male/female (eyes)	56/74	26/29	NS^‡^
Lens status			
Preserved (phakic)	4	0	NS^‡^
PEA + IOL	116	46	
Pseudophakic	10	9	
Iatrogenic RB	48 (36.9%)	7 (12.7%)	0.0008^‡^
Postoperative RD	4 (3.1%)	4 (7.3%)	NS^‡^
Preoperative PVD	41 (31.5%)	15 (27.3%)	NS^‡^
Lattice degeneration	19 (14.6%)	4 (7.3%)	NS^‡^

^*∗*^Mean ± standard deviation;^†^unpaired *t*-test; ^‡^Fisher's exact probability test; comparisons between surgery groups; NS: not significant; PEA + IOL: phacoemulsification and intraocular lens implantation; PVD: posterior vitreous detachment; RD: retinal detachment; RB: retinal break.

**Table 2:** Comparison of the incidence of iatrogenic retinal breaks with or without preoperative posterior vitreous detachment and with or without lattice degeneration.

	20 gauge (130 eyes)	25 gauge (55 eyes)	*P* value
PVD (+)	6/41 (14.6%)	3/15 (20.0%)	NS^*∗*^
PVD (−)	42/89 (47.2%)	4/40 (10.0%)	0.0001^*∗*^
*P* value	0.0007^†^	NS^†^	
LD (+)	15/19 (78.9%)	3/4 (75.0%)	NS^*∗*^
LD (−)	33/111 (29.7%)	4/51 (7.8%)	0.021^*∗*^
*P* value	0.0001^†^	0.002^†^	

LD: lattice degeneration (+ present; − absent); PVD: posterior vitreous detachment (+ present; − absent); Fisher's exact probability test: ^*∗*^comparison between surgery types within each pathological condition; ^†^comparison within surgery type, between eyes with and without other pathological conditions; NS: not significant.

## Data Availability

The data used to support the findings of this study are available from the corresponding author upon request.
